# A year later

**DOI:** 10.1590/0100-6991e-2021EDIT01

**Published:** 2021-03-05

**Authors:** Maria isabel Toulson Davisson Correia

**Affiliations:** 1 - Universidade Federal de Minas Gerais, Departamento de Cirurgia - Belo Horizonte - MG - Brasil

## EDITORIAL

Twenty-twenty has been a year of apprehension, fears, adversities, changes, adaptations, and challenges. It will be forever present in everyone’s memory, defined not only by all the previous words but also by having been tackled by a minuscule living being - Covid19! Furthermore, conspiration theories, fake news, pessimism, political disputes, and poverty increase have also imprinted the world history, while concomitantly, there has been a frenetic race led by the scientific community to develop the saver vaccine against the Sars-Covid19. 

In the middle of the whirlwind, Science has been questioned and lost its balance. On one side, some people believe in the relevancy and veracity of adequate methodologic data, and on the other, those who ignore them. However, it is under adversities that the human beings seem to reinvent themselves, and perspectives, many times rather challenging bloom, contradicting the “blindness” of those who cannot give foresee some light. As stated by Saramago - the apprentice thought: “we are blind,” and he sat down to write Blindness to remind those who might one day read it that we perversely use reasoning when humiliating life, that the human being dignity is daily insulted by the mighty influential people in our world, that the universal lies have taken over the plural trues, that Man has lost his self-respect when he lost the respect towards his fellow-creature^1^. Nevertheless, there is hope!

Among this contemporary fussiness, the Journal of the Brazilian College of Surgeons has faced similitudes. For us, from the new Editorial Board, the challenge has been Herculean, as, beyond the “novelty,” the number of submitted papers has increased by 30% in 2020. That has required a more rigorous review process. After all, it is not enough to have many manuscripts; it is also fundamental to have minimum quality criteria so that these papers might later be cited, and therefore might contribute to the impact factor. 

The impact factor should not be the only premise when assessing scientific publications, mainly when it has been the reason for many controversies and discussions^2^. Undoubtedly, there are other aspects to be considered, particularly when the journal belongs to a scientific society as the Brazilian College of Surgeons (CBC). One of CBC’s pillars is knowledge sharing, and the journal is one of its examples. However, worldwide, research funding agencies use metrics and assess investigators based on their publication history contemplating the impact factor. Therefore, it is impossible to deny its relevance. 

In 2020, the CBC journal acceptance rate decreased to 27% versus 38% in 2019, and it may remain around this cutoff or even lower. Scientific quality and rigor should set the tone; although this is seldom discussed in Brazil, it must be tackled. Thus, to reach quality in the editorial process, we have decided to rely on distinct surgical experts; these are the associate editors. 

The associate editors’ selective process was democratically open to all CBC members who were required to fulfill pre-defined criteria to be part of such a challenging task. It was with great pleasure and surprise that we saw many surgeons interested in carrying it out. Therefore, currently, the CBC journal has nine associate editors who work in syntony with the editor in chief and with the other members of the editorial board or the ad hoc invited reviewers. We hope to reach new goals, embarking on the principles of scientific quality publications, in a country where it is a tremendous challenge to carry out research. However, it is plausible to conduct local studies, even as they may seem “simple” once the scientific method percepts are respected and correct. Such manuscripts will undoubtedly be of great benefit to the practice of medicine in the country and to our patients. It is mister to stimulate the correct method! 

A rigorous scientific method and appropriate language are essential to reach credibility and applicability in research. However, such a premise is rarely taught in graduation and post-graduation courses, negatively impacting what is published worldwide^3^ ^-^ ^5^. According to Ioannidis^4^, the chance the primary goal (question) of a scientific study be real depends on the statistical power and bias. Moreover, considering that most of the studies have small sample sizes and that a large number does not even mention this basic/essential percept, the results may be absolutely questionable due to type I or II statistical errors. Furthermore, with the currently predatory publishing industry^6^ ^,^ ^7^, publishing has become an easy task, impacting clinical practice. 

Someone who is not aware of the publication healthcare industry believes that everything published should be taken as a conduct determinant^8^. Science has changed the world, but we need to start thinking about changing Science by being more critical and less complacent with everything presented. In this regard, the last year has also been marked by scientific paper retractions in internationally renowned journals, which has negatively impacted patient care by inadequate translation of questionable and non-sound papers^9^. 

In the core of the scientific discussion, negative human characteristics are often seen, such as vanity and lack of ethics, perpetuated and not challenged by non-critical readers. For the latter, whatever is published in journal X or Y that has an “impact factor” is enough. Once again, it is fundamental to acknowledge the role of the scientific method^10^, justifying the efficacious translation of its principles. On the other hand, the speed of knowledge translation has been high, particularly when sensationalist and “in fashion” topics are covered, to the detriment of those of utmost importance to the spreading of adequate data interpretation. The latter is extremely relevant to humanity. The high number of Pubmed indexed publications, which in 2009 accounted for 1000/day^11^, does not imply good Science since many have inadequate scientific methods^4^ ^,^ ^5^ ^,^ ^12^. It is imperative to be critical!

The world gallops fast, and the need for quick publications is required; this is to say, decreased time between submission and publication is an essential determinant of journal quality. However, the whole publication pathway demands the careful fulfilling of steps, which comprise not only quality of the submitted paper, adequate peer-review and editorial decision but also a complicated logistic. The latter requires elaborated organizational systems. Unfortunately, there are flaws once the control of all the steps surpasses the editorial board’s responsibility. It is an interprofessional task that is associated to costs and political relationships non-related to the editors’ competencies. In this regard, this will remain a considerable challenge, mainly because the pandemic economic sequela has become prevalent. Let us move on!

As the “captain” of this editorial board, the goals and metrics will always be linked to offer ethical scientific information, bearing in mind that plagiarism must be early identified^13^; provide the best available quality manuscripts while understanding the limitations of research in a country with scarce financial resources and where scientific knowledge still crawls, despite its tremendous potential to impact patient care and; positively disseminate adequate knowledge to professionals of this continental Brazil. After all, the leading motto for those who work in the healthcare sector is good care of patients!
